# The impact of COVID-19 and strategies for mitigation and suppression in low- and middle-income countries

**DOI:** 10.1126/science.abc0035

**Published:** 2020-06-12

**Authors:** Patrick G. T. Walker, Charles Whittaker, Oliver J. Watson, Marc Baguelin, Peter Winskill, Arran Hamlet, Bimandra A. Djafaara, Zulma Cucunubá, Daniela Olivera Mesa, Will Green, Hayley Thompson, Shevanthi Nayagam, Kylie E. C. Ainslie, Sangeeta Bhatia, Samir Bhatt, Adhiratha Boonyasiri, Olivia Boyd, Nicholas F. Brazeau, Lorenzo Cattarino, Gina Cuomo-Dannenburg, Amy Dighe, Christl A. Donnelly, Ilaria Dorigatti, Sabine L. van Elsland, Rich FitzJohn, Han Fu, Katy A. M. Gaythorpe, Lily Geidelberg, Nicholas Grassly, David Haw, Sarah Hayes, Wes Hinsley, Natsuko Imai, David Jorgensen, Edward Knock, Daniel Laydon, Swapnil Mishra, Gemma Nedjati-Gilani, Lucy C. Okell, H. Juliette Unwin, Robert Verity, Michaela Vollmer, Caroline E. Walters, Haowei Wang, Yuanrong Wang, Xiaoyue Xi, David G. Lalloo, Neil M. Ferguson, Azra C. Ghani

**Affiliations:** 1 MRC Centre for Global Infectious Disease Analysis, Department of Infectious Disease Epidemiology, Imperial College London, London, UK.; 2 Pathology and Laboratory Medicine, Warren Alpert Medical School, Brown University, Providence, RI, USA.; 3 Department of Infectious Disease Epidemiology, London School of Hygiene and Tropical Medicine, London, UK.; 4 Department of Statistics, University of Oxford, Oxford, UK.; 5 Liverpool School of Tropical Medicine, Liverpool, UK.

## Abstract

The ongoing coronavirus disease 2019 (COVID-19) pandemic poses a severe threat to public health worldwide. We combine data on demography, contact patterns, disease severity, and health care capacity and quality to understand its impact and inform strategies for its control. Younger populations in lower-income countries may reduce overall risk, but limited health system capacity coupled with closer intergenerational contact largely negates this benefit. Mitigation strategies that slow but do not interrupt transmission will still lead to COVID-19 epidemics rapidly overwhelming health systems, with substantial excess deaths in lower-income countries resulting from the poorer health care available. Of countries that have undertaken suppression to date, lower-income countries have acted earlier. However, this will need to be maintained or triggered more frequently in these settings to keep below available health capacity, with associated detrimental consequences for the wider health, well-being, and economies of these countries.

## Global prospects for COVID-19 control

Lower-income countries have recognized the potential impact of coronavirus disease 2019 (COVID-19) from observing ongoing epidemics. Many have intervened quickly and early with measures to slow viral transmission, which may partly explain the low rates observed so far in these countries. Walker *et al.* calibrated a global model with country-specific data (see the Perspective by Metcalf *et al.*). Despite the potentially protective effects of younger demographics, the closer intergenerational contact, limitations on health care facilities, and frequency of comorbidities in lower-income countries require sustained nonpharmaceutical interventions (NPIs) to avoid overwhelming health care capacity. As a result of strict NPIs, the protective effects of immunity will be reduced, and it will be important to improve testing capacity. Ensuring equitable provision of oxygen and—when they are ready—pharmaceutical interventions should be a global priority.

*Science*, this issue p. 413; see also p. 368

## Teaser

Modeling reveals differences in the unfolding COVID-19 epidemics and responses to their control among countries with different income levels.

The coronavirus disease 2019 (COVID-19) pandemic caused by the severe acute respiratory syndrome coronavirus 2 (SARS-CoV-2) virus is a major global health threat, with 5.4 million cases and 344,000 deaths confirmed worldwide as of 26 May 2020 (*1*). The experience in countries to date has emphasized the intense pressure that a COVID-19 epidemic places on national health systems, with demand for intensive care beds and mechanical ventilators rapidly outstripping their availability, even in relatively highly resourced settings (*2*). This has potentially profound consequences for resource-poor settings, where the quality and availability of health care and related resources (such as oxygen) is typically poorer (*3*). We sought to understand the factors that could result in a differential impact of the COVID-19 pandemic in low- and middle-income countries (LMICs), as well as to evaluate the potential strategies for suppression and mitigation in these settings given the current global state of the pandemic.

## Demography and social contact patterns

We collated data on global demographic projections of population size by age and country and available data on social mixing patterns by age and country-level income category. We first include these within a simple SIR modeling framework (*4*) to estimate the theoretical final size of the outbreak (age-specific attack rate) in the absence of nonpharmaceutical interventions (NPIs). To illustrate how these would determine the demand for health care over the course of an unmitigated epidemic, we applied age-specific estimates of the rates of hospitalization and of the proportion of these requiring critical care, and of the infection fatality ratio (IFR) (*5*) under an initial assumption of a consistent underlying role of comorbidities and the same level of medical care supplied during the epidemic in China (see materials and methods). On the basis of the observed doubling time in the incidence of deaths across Europe (*6*), we use a central estimate of the basic reproduction number (*R*_0_) of 3.0 (a 3.5-day doubling time) and investigate scenarios with *R*_0_ between 2.3 (a 5-day doubling time) and 3.5 (a 3-day doubling time).

**Fig. 1:**
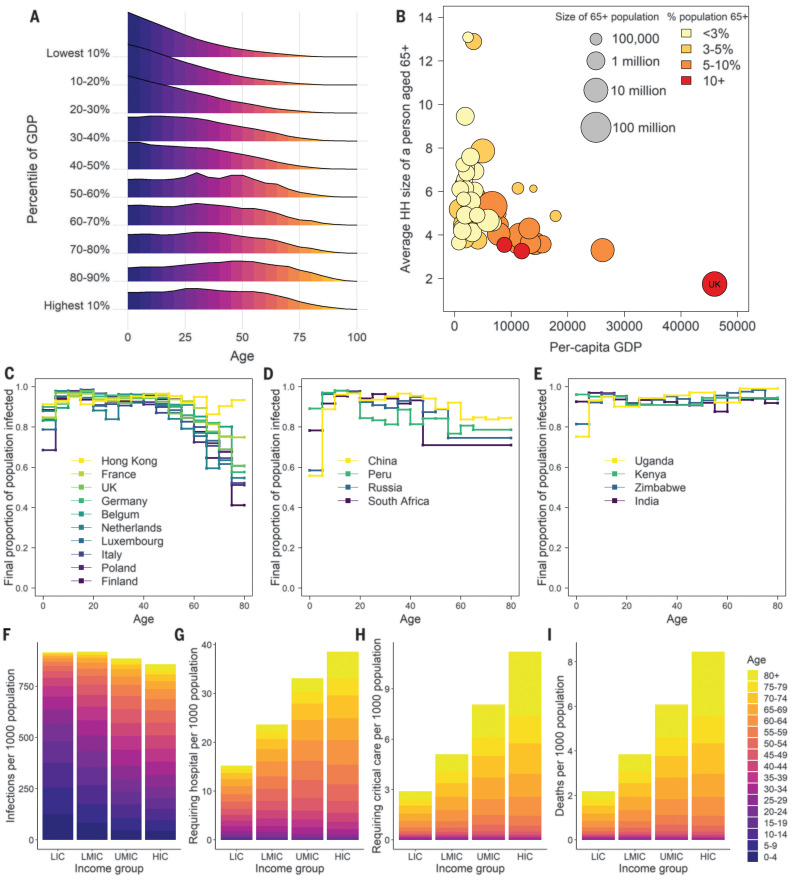
Demographic, societal, and mixing patterns relevant to SARS-CoV-2 transmission and burden. (**A**) Aggregated demographic patterns within 2020 World Population Prospects projections across countries within each 2018 World Bank GDP per-capita decile. (**B**) Average household (HH) size within Demographic Health Surveys of individuals aged 65 and over by 2018 World Bank GDP per capita. For reference, the average household size of contacts in the UK is also provided as an example for a HIC. (**C**) Final proportion of population infected in an unmitigated epidemic for an age-structured SIR model with *R*_0_ = 3.0 and age-specific social mixing based on contact surveys identified in HICs. (**D** and **E**) Equivalent figure for surveys identified in UMICs and LMICs/LICs, respectively. (**F** to **I**) Output from simulations across countries of an unmitigated pandemic with *R*_0_ = 3.0. (F) Attack rate in terms of number of individuals infected per 1000 population. (G) Equivalent rates of infection leading to illness requiring hospitalization. (H) Illness requiring critical care. (I) Mortality assuming a health system functioning at the level of China throughout the pandemic. LIC, low-income country; LMIC, low- and middle-income country; UMIC, upper–middle-income country HIC, high-income country.

Figure 1 summarizes two of the demographic and societal factors that are likely to determine the burden of COVID-19 disease across different income settings. First, there is a strong correlation between the gross domestic product (GDP) of a country and its underlying demography (Fig. 1A). High-income countries (HICs) tend to have the oldest populations; low-income countries (LICs), by contrast, have a much smaller proportion of the population who are above age 65 and therefore within the age interval currently observed to be at particularly high risk of mortality from COVID-19 disease (*5*). Second, the household is a key setting for SARS-CoV-2 transmission (*7*). The average size of households that have a resident over the age of 65 years is substantially higher in LICs (Fig. 1B) compared with middle-income countries (MICs) and HICs, increasing the potential for spread generally but also specifically to this particularly vulnerable age group. Contact patterns between age groups also differ by country (fig. S5); in high-income settings, the number of contacts tends to decline steeply with age. This effect is more moderate in middle-income settings and disappears in low-income settings, indicating that elderly individuals in these settings (LICs and MICs) maintain higher contact rates with a wider range of age groups compared to elderly individuals in HICs. These contact patterns influence the predicted SARS-CoV-2 infection attack rate across age groups (Fig. 1, C to E), with higher attack rates in the elderly predicted in low-income settings compared to high-income settings and middle-income settings showing intermediate patterns.

For an unmitigated epidemic, we obtain similar estimates of the distribution of the attack rate across settings for a given *R*_0_ (Fig. 1F), with slightly higher attack rates in LICs due to the more homogeneous levels of mixing with age. However, under a baseline assumption of the same comorbidity profile across all settings, we would expect a lower risk of requiring hospitalization and critical care in lower-income settings, driven by the younger demography in these populations (Fig. 1, G to H). Assuming the same availability of health care (equivalent to that provided in China) throughout the pandemic, we would expect a lower overall per-capita risk of mortality in lower-income settings owing to the younger age of the populations (Fig. 1I).

## Health care availability and quality

**Fig. 2:**
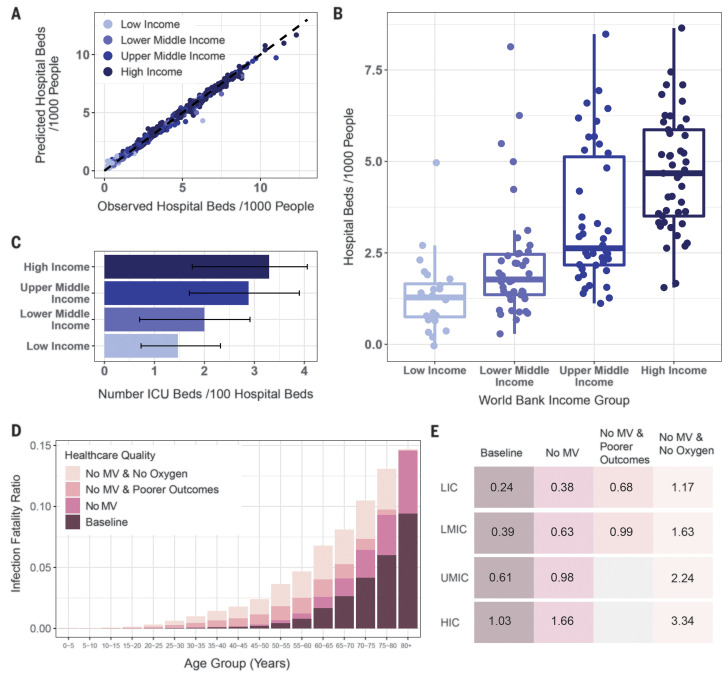
Estimates of hospital bed and ICU capacity, and the potential impact of health care quality on the IFR. (**A**) Comparison of BRT model prediction and empirically observed numbers of hospital beds per 1000 population. Each point represents a country, with the *x* axis indicating the observed number of hospital beds per 1000 population for that country and the *y* axis indicating the model-predicted number of hospital beds per 1000 population. Coloring of the points indicates which World Bank income strata the country belongs to. (**B**) Boxplots of the number of hospital beds per 1000 population, stratified by World Bank income group. Points are modeled estimates of hospital beds per 1000 population obtained from the model. (**C**) Results from a systematic review describing the percentage of all hospital beds that are in ICUs, stratified by World Bank income group. Error bars indicate the interquartile range of the median. (**D**) Age-stratified scenarios for the IFR under different health care quality. The baseline age groups are estimates based on data for high-income settings. “No MV” denotes not being able to access an ICU unit with mechanical ventilation available. “Poorer outcomes” represents a higher risk of mortality from severe pneumonia in an LMIC setting if only limited or poor-quality oxygen support is available. “No Oxygen” represents the outcomes if hospitalized patients do not receive oxygen support. The stacked bars represent the cumulative increase in IFR at each stage. Note that the final stage “No MV and No Oxygen” represents the additional IFR due to increasing mortality rates from 20% in the presence of limited or poor-quality oxygen support to 60% in the absence of any oxygen support. (**E**) Estimated representative IFR averaged across age groups in different settings under a range of health care quality assumptions. The differences between LIC, LMIC, UMIC, and HIC at baseline reflect the demography and social contact patterns but otherwise assume the same health care quality. Lower health care quality is not shown for UMIC and HIC as these settings are likely to have the quality of health care incorporated in the baseline estimates.

It is clear from the current epidemics in Europe and the United States that COVID-19 disease will place a severe strain on health systems. This effect is likely to be more extreme in lower-income settings where health care capacity is typically limited. To explore this conjecture, using data derived from the World Bank and wider literature, we developed a model of the supply of health care relevant to COVID-19 disease. We used a boosted regression tree (BRT)–based approach to model the likely availability of hospital beds (per 1000 population, from the World Bank) on the basis of a suite of relevant health care–related and socioeconomic covariates (also from the World Bank; see materials and methods). This prediction of hospital bed capacity was then combined with estimates of intensive care unit (ICU) beds (per 100 hospital beds) across a range of different settings [spanning LICs, LMICs, upper–middle-income countries (UMICs), and HICs] identified through a systematic literature review (Fig. 2). These estimates of health care capacity were then integrated with our estimates of the demand that COVID-19 epidemics will place on national health systems.

The boosted regression tree model predicts hospital bed capacity well across the range of countries for which data were available (Fig. 2A). We find that hospital bed capacity is strongly correlated with the income status of countries (Fig. 2B); LICs have the fewest hospital beds per 1000 population (median 1.28 beds per 1000) and HICs the highest (median 4.68 beds per 1000 population). LMICs and UMICs fall between these two extremes (1.77 and 2.63 beds per 1000 population on average, respectively). We find that the percentage of hospital beds that are in ICUs is lowest in LICs (1.47% on average) and highest in HICs (3.30%), with LMICs and UMICs falling in-between (2.00 and 2.88%, respectively) (Fig. 2C). Our estimates of the ICU capacity in HICs are drawn almost exclusively from a recent review of ICU capacity in Asian countries (*8*) and are not therefore necessarily reflective of ICU capacity in HICs worldwide.

To understand the potential impact of weaker health systems on the IFR, we collated expert clinical opinion on the likely outcomes for COVID-19 patients (materials and methods). These expert estimates drew on recent experience treating COVID-19 patients in the United Kingdom (UK) alongside an understanding of severe pneumonia outcomes in LIC and LMIC settings. Mechanical ventilation (MV), which has been required by 80% of COVID-19 ICU patients in the UK (*9*), is of very limited capacity in many LIC and LMIC countries. Across sub-Saharan Africa, for example, recent estimates put the average number of ventilators at only 172 per country (*10*). In the absence of MV, the consensus was that the mortality rate would be in the range of 90 to 100%. This compares to a mortality rate of 51.6% in COVID-19 ICU patients who require MV in the UK (*9*). Similarly, for the 20% of individuals who would be admitted to the ICU in the UK but not require MV, the consensus was that mortality would be 50 to 65% in an LIC or LMIC setting. It was, however, noted that there would likely be considerable heterogeneity in this rate (not captured here) because of the variation in both the quality of hospital care and availability of hospital facilities within and between countries (with better facilities concentrated in urban areas and capital cities compared to rural areas). For individuals with severe pneumonia requiring hospitalization, mortality rates are expected to be higher in LICs and LMICs than in HICs. The values for LICs and LMICs assumed here are a mortality rate across all age groups of 20 to 30% if oxygen support is available, while anticipating that this may not be at sufficiently high flow directly at the bedside to ensure outcomes comparable to those in HICs (“Poorer Outcomes” in Fig. 2), and 60% if oxygen support is not available (because of health care capacity being exceeded). Using these parameters, we expect a larger proportion of deaths to occur in those aged 40 and upward in LIC and LMIC settings (Fig. 2D) and that the lack of quality oxygen support will disproportionately increase mortality in younger age groups (as these age groups are more likely to require oxygen support than MV). Notably, the lack of health system capacity is likely to increase the overall IFR in LIC and LMIC settings, offsetting the apparent protective effects of the younger population (Fig. 2E). In our subsequent modeling, we therefore examined three scenarios for LICs and LMICs to examine the impact of health care quality and quantity upon potential COVID-19 burden: a scenario in which there were no health care constraints and quality of care was similar to that of HICs (“Unlimited health care”); a scenario using typical health care constraints on hospital and ICU beds such as those shown in Fig. 2, B and C (“Limited health care”); and a scenario in which there is an assumed absence of MV and treatment for severe pneumonia is less effective (“Limited health care, No MV and poorer outcomes”).

## Comorbidities

**Fig. 3:**
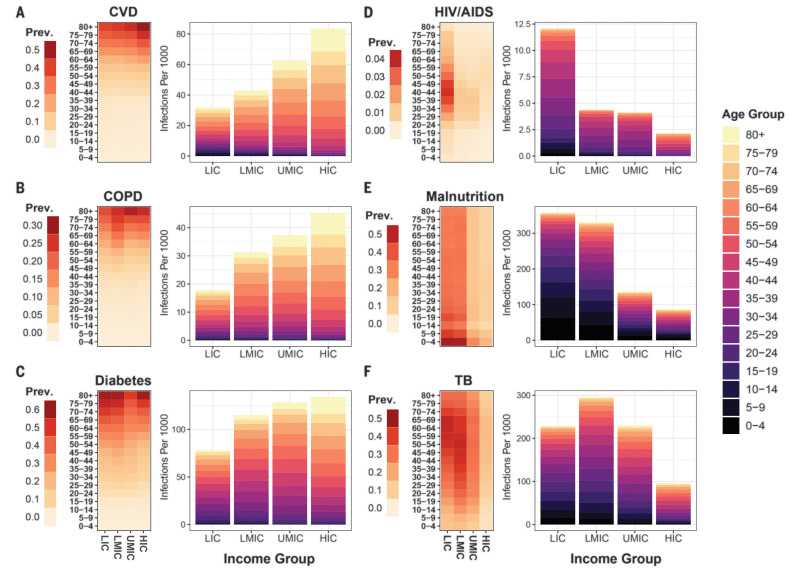
The prevalence of different comorbidities across income settings and the proportion of SARS-CoV-2 infections co-occurring with them. The age distribution of comorbidities relevant as modifiers of COVID-19 disease severity was extracted from Global Burden of Disease 2017 estimates (*12*) and integrated with estimates of the predicted age distribution of infection in an unmitigated pandemic scenario. For (**A**) cardiovascular disease (CVD), (**B**) chronic obstructive pulmonary disease (COPD), (**C**) diabetes, (**D**) HIV/AIDS, (**E**) malnutrition, and (**F**) tuberculosis, the left heatmap shows the age distribution of these comorbidities across different income settings, expressed as the proportion of the population in that income setting that has the comorbidity. The bar charts (colored according to age group) show the number of infections per 1000 population that co-occur with the respective comorbidity.

There remain large uncertainties in the underlying determinants of the severity of SARS-CoV-2 infection and how these translate across settings. However, clear risk factors include age (*5*) and underlying comorbidities that include hypertension, diabetes, coronary vascular disease (CVD), and chronic obstructive pulmonary disease (COPD), which serve to exacerbate symptoms (*11*). The prevalence of these conditions varies substantially across populations and by age (Fig. 3). Using Global Burden of Disease 2017 estimates (*12*), our unmitigated scenario leads to 6.1, 3.8 and 13.3% of SARS-CoV-2 infections occurring in individuals with CVD, COPD, and diabetes, respectively.

In LICs and LMICs, there is a higher burden of infectious diseases such as HIV/AIDS and tuberculosis (TB), and of poverty-related determinants of poorer health outcomes such as malnutrition, than in HICs. These generally occur in younger populations (Fig. 3, D to F). Although infectious diseases and malnutrition are not yet recognized as specific risk factors for poor prognosis from COVID-19, owing to a lack of data from settings in which they are prevalent, it is possible that the risk profile in LIC and LMIC settings will be very different from that observed to date in China, Europe, and North America. Understanding the extent to which these potential comorbidities make younger populations more vulnerable to severe sequalae of COVID-19 and designing strategies to protect them will be important in adapting pandemic responses to lower-income countries.

## Mitigation and suppression strategies for low- and middle-income countries

To understand the consequences of the demographic, social contact, and health system patterns on strategies for reducing the spread of SARS-CoV-2 in the context of control measures already enacted by countries, we developed an age-structured SEIR modeling framework to explore the dynamics of the epidemic under different health system capacity constraints (see materials and methods). As for the SIR final size calculations, we use demographic data and social contact patterns based on representative settings in the LIC, LMIC, UMIC, and HIC strata. Disease progression for individuals requiring hospitalization was explicitly modeled to track requirements for hospital bed provision for severe pneumonia (those for whom we assume oxygen support will be required) and for ICU provision [those for whom we assume 80% would require MV in line with U.K. data (*9*)]. Durations of stay were based on U.K. data (*9*, *13*); although it is likely that these will be shorter in LIC and LMIC countries, in the absence of data to guide these parameters, the results presented here are conservative (i.e., hospital capacity will be exceeded earlier). Although health care–seeking behavior may also differ across settings, we make the simplifying assumption that all symptomatic cases seek care. To capture the uncertainty in hospital demand, we generated 500 parameter sets drawing on uncertainty in key parameters determining the probability of hospitalization, of requiring critical care, and of outcomes (death or recovery) in each hospital state and ran 10 stochastic realization using each parameter set (see materials and methods). The 95% range across the simulations is presented as an uncertainty interval (UI). We consider two potential strategies [similar to those previously illustrated for pandemic influenza planning and in the early stages of the COVID-19 epidemic (*14*, *15*)]: (i) mitigation, whereby transmission is reduced but *R*_t_ remains above 1 and hence a single-peaked epidemic is predicted owing to the buildup of herd immunity; and (ii) suppression, whereby transmission is reduced such that *R*_t _< 1 and hence, if interventions are later released, transmission will be expected to rise as herd immunity will not have been achieved.

We used our final SIR size calculations to estimate the degree of social distancing that results in “optimal” mitigation. This is defined as the maximum reduction in transmission that can be achieved if a uniform reduction in contact rates is implemented at the start of the epidemic for an undefined but finite period such that *R*_t _~1 and a single-peaked epidemic is generated (see materials and methods). If “optimal” mitigation based on enhanced social distancing is pursued, for an *R*_0_ of 3.0, we estimate a maximum reduction in infections in individual countries in the range 30 to 38% (median 33%) and a range of reduction in mortality between 19 and 55% (median 39%) (assuming the mortality patterns observed in China). These optimal reductions in transmission and burden were achieved with a range of reductions in the overall rate of social contact across countries between 40.0 and 44.9% (median 43.9%), with this range across countries increasing to 42.9 to 47.9% (median 46.9%) for an *R*_0_ of 3.5 and decreasing to 31.4 to 35.8% across countries (median 35.0%) for an *R*_0_ of 2.3. Combining mitigation with enhanced social distancing of elderly individuals is predicted to result in greater mortality reductions of 23 to 67% across countries (median 49%) for *R*_0 _= 3 (table S5). However, both of these strategies are predicted to have a lower proportional impact in lower-income settings compared to higher-income settings: median reductions in mortality in the range of 19.5 to 41.6% (median 25.3%) in LICs, in contrast to 21.5 to 55.1% (median 49.9%) in HICs for optimized mitigation strategies including social distancing, and in the range of 25.4 to 50.9% (median 32.6%) in LICs in contrast to 23.4 to 66.6% (median 60.1%) in HICs for optimized mitigation strategies including enhanced social distancing for elderly individuals (table S5). This lower proportional reduction in deaths of mitigation scenarios in lower-income settings is driven by the more homogeneous contact patterns by age in these settings (Fig. 1), resulting in more persistent spread to older age categories as contact rates in the general population are reduced (fig. S6).

**Fig. 4:**
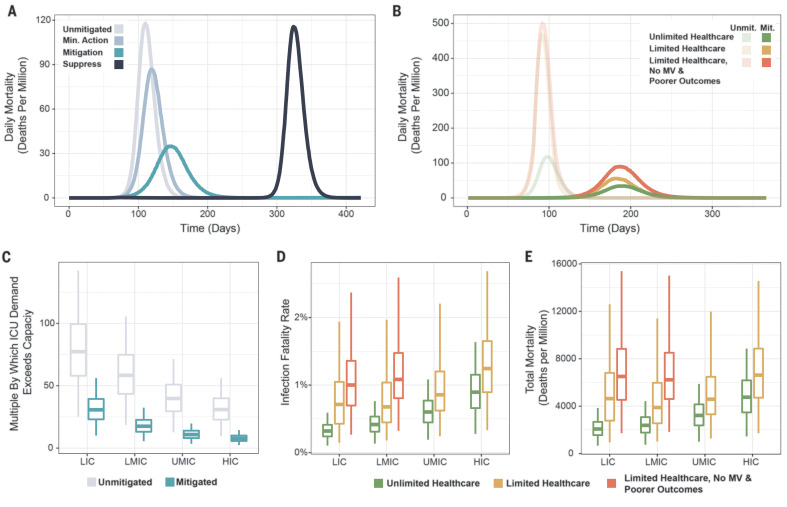
The impact of health care capacity and quality on COVID-19 mortality in different settings. (**A**) Representative epidemic trajectories for an unmitigated epidemic (gray line), an epidemic involving minimal social distancing (pale blue line, 20% reduction in social contacts), an epidemic involving extensive social distancing (teal, 45% reduction in social contacts), and an epidemic trajectory that involves extensive suppression (75% reduction in social contacts) followed by lifting of restrictions after 6 months, leading to resurgence (dark blue line). (**B**) The excess deaths associated with constraints on health care quality and quantity, including the deaths associated with a hypothetical setting with unlimited high-quality health care (green lines), settings where high-quality health care is available but limited (yellow lines), and settings where only limited, poorer-quality health care is available (orange lines). Pale lines show an unmitigated scenario, colored lines a mitigated scenario. (**C**) The multiple by which ICU demand exceeds capacity for each World Bank income strata for an unmitigated (gray) and mitigated (teal) epidemic. (**D**) The modeled IFR for different World Bank income strata under different scenarios of health care quality and quantity available, assuming a mitigated scenario in which baseline contacts are reduced by 45%. (**E**) The modeled deaths per million population for different World Bank income strata under different assumptions of health care quality and quantity available, assuming a mitigated scenario in which baseline contacts are reduced by 45%. Plots show medians (bars) and interquartile ranges (boxes), as well as points <1.5× the interquartile range (whiskers) and >1.5× (points) from 500 parameter draws.

Figure 4 highlights the dynamical impact of different control measures on COVID-19 epidemics. Scenarios in which in which the COVID-19 epidemic is suppressed for a period of 6 months before returning to prepandemic social contact patterns leads to rapid resurgence of the virus and a delayed peaking epidemic (Fig. 4A). There is a marked reduction in disease burden under the optimal mitigation strategy, which results in a single-peaked epidemic with a substantially lower peak compared to the unmitigated epidemic. However, this single peak relies upon the assumption that recovery from infection confers durable immunity to reinfection, which has yet to be conclusively demonstrated (*16*). Furthermore, although “optimal” mitigation is the strategy that will minimize infections and achieve herd immunity in a single-peaked epidemic, there are multiple other strategies that can also minimize infections over a longer term (*17*, *18*). Any mitigation scenario will also always be worse in terms of both the peak hospital demand and total predicted deaths than scenarios in which the epidemic is suppressed (i.e., the reproduction number over time, *R*_t_, is kept below 1) (Fig. 4, A and B). However, if suppression cannot be successfully maintained, then a delayed epidemic may occur, which may outweigh the benefits of the original suppression strategy and result in higher mortality than if a mitigation scenario had been successfully pursued. Such a second peak is not inevitable—wide-scale suppression may provide countries with the time to develop testing and contact tracing systems, as well as locally targeted responses, that can help to maintain lower levels of transmission once the initial suppression interventions are relaxed. It is important to note that our framework does not currently provide insights into the specific combinations of NPIs required to achieve such reductions, and these are likely to differ across settings according to various factors such as school attendance and occupation. Other factors such as reduced ability to work from home and general economic vulnerability will affect the abilities of populations to adhere to stringent NPIs that involve restrictions on movement. Meanwhile, larger household sizes, and subsequently higher levels of household-based transmission, may limit the impact of self-isolation and increase the social and economic impact of self-isolation measures in lower-income settings.

The comparative benefits and drawbacks of these scenarios (in terms of direct health impact of COVID-19 disease) will differ between settings depending on their health care capacity and quality. In all settings, although our optimized mitigation scenario is predicted to substantially reduce the gap between demand for hospital beds and capacity, demand for critical care is still predicted to vastly exceed capacity, leading to a substantial additional burden relative to a scenario with unlimited capacity (Fig. 4B). Although we predict lower demand for critical care in lower-income settings because of their younger populations, this is likely to be offset by a much lower level of supply: For our mitigation scenario, including population-level social distancing, peak demand for critical care in our simulation for a typical LIC outstrips supply by a factor of 30.7 (95% UI 14.7–48.8), whereas for the equivalent simulation in a typical HIC this factor was 7.8 (95% UI 3.6–13.0) (Fig. 4C). Typical LMICs and UMICs produced factors of overdemand of 17.5 (95% UI 8.3–28.6) and 10.9 (95% UI 5.1–17.7) respectively.

We estimate that constraints on health care capacity would be likely to increase the IFR under a mitigation strategy in all settings. However, as has been observed, HICs and UMICs are likely to be able to put in place surge capacity to limit any impact on mortality (*19*–*21*). By contrast, in LICs and LMICs, we predict that the poorer quality of health care available is likely to have a greater impact on the overall IFR than the limits on capacity alone. If the health care quality in these settings was at the same level as in HICs and not subject to capacity constraints, we estimate 2.1 (95% UI 1.0–3.3) deaths per 1000 population in an LIC and 2.4 (95% UI 1.1–3.9) deaths per 1000 population in an LMIC. This increases to 4.6 (95% UI 1.4–10.0) and 3.9 (95% UI 1.5–8.9) deaths per 1000 population with health care capacity limits, in LICs and LMICs, respectively, and to 6.5 (95% UI 2.7–12.3) and 6.2 (95% UI 2.8–11.9) deaths per 1000 population, respectively, if the poorer-quality health care is also factored in. Overall, this represents 4.4 (95% UI 1.7–9.0) and 3.8 (95% UI 1.7–8.0) excess deaths per 1000 population due to both the poorer-quality health care and lack of health care capacity in LICs and LMICs, respectively.

## Suppression and longer-term exit strategies

Almost all countries and territories have now reported at least one COVID-19 case, with many now also reporting deaths. Although individual countries have responded differently to this threat, most have implemented some form of NPI to either mitigate the burden of the epidemic or to suppress transmission (*22*, *23*). We reviewed data on the interventions that had been collated within the ACAP COVID-19 Government Response Measures dataset to summarize the stage of the epidemic at which countries have implemented suppression measures (*23*) (see materials and methods).

Table 1 summarizes the stage of the epidemic at which those countries implementing suppression measures did so (see supplementary materials for country-level analysis). Across the different regions, countries in Europe and Central Asia have initiated suppression measures at a later stage of their epidemics (in terms of per-capita cases and deaths) than other regions to date. This may be due partly to censoring (i.e., other countries have yet to impose suppression measures since the date the dataset was downloaded on 20 April 2020), as well as to the wider recognition of the potential impact of COVID-19 that countries in other regions can observe from the ongoing epidemics in Europe. However, there is also a strong gradient in the timing of lockdowns with income status, with LICs and LMICs initiating suppression measures earlier than UMICs or HICs.

**Table 1:** Estimated stage of the epidemic at suppression across regions and income strata and ratios of reported cases to deaths prior to suppression.

	Countries initiating suppression measures*	**Median date suppression implemented** **(range)**	**Median cases/million prior to suppression (range)**	**Median deaths/million prior to suppression (range)**	**With ≥3 deaths prior to suppression†**	**Median ratio of cases to deaths**
Worldwide	121	24/03 (08/03–19/04)	6.01 (0–449.24)	0 (0–10.447)	50	45.8 (5.1–325.2)
**Region**						
East Asia and Pacific	10	25/03 (14/03 – 10/04)	3.08 (0–149.37)	0 (0–0.549)	9	47.3 (9–325.2)
Europe and Central Asia	34	22/03 (08/03– 03/04)	72.08 (0.07–449.24)	0.340 (0–10.447)	20	46.7 (9–320.3)
Latin America and Caribbean	21	22/03 (16/03– 08/04)	6.01 (0.18–156.59)	0 (0–0.393)	6	63.9 (30.2–102)
Middle East and North Africa	14	23/03 (17/03–30/03)	12.44 (0–353.31)	0 (0–1.763)	6	16.5 (5.1–139.7)
North America	0	NA‡	NA	NA	2	43.5 (14.5–72.6)
South Asia	4	26/03 (20/03–02/04)	2.41 (0.07–35.15)	0 (0–0.051)	1	80 (NA)
Sub-Saharan Africa	38	28/03 (21/03–19/04)	0.92 (0–101.68)	0.002 (0–1.799)	5	10.7 (7.8–63.6)
**Income strata**						
Low income	23	28/03 (20/03–19/04)	0.57 (0–6.13)	0 (0–0.791)	3	8.6 (7.8–10.7)
Lower middle income	22	25/03 (14/03–10/04)	0.58 (0.05–30.99)	0 (0–1.799)	7	19.3 (9–80)
Upper middle income	41	24/03 (16/03– 08/04)	9.98 (0–135.34)	0.044 (0–3.548)	13	38.8 (5.1–191.8)
High income	35	23/03 (08/03–08/04)	97.30 (5.14–449.24)	0.202 (0–10.447)	27	72.6 (9–325.2)

*Does not include countries implementing suppression not listed in ACAP data as of 17 April 2020.

†Includes countries that have not yet implemented suppression in ACAP data as of 17 April 2020 where infections are assumed to have grown exponentially until the date of the most recent measure reported in ACAP. For countries excluded from suppression analysis (Brazil, China, Iran, Japan, Singapore, South Korea, and the United States of America), analysis was restricted to the time before the 10 cumulative deaths reported.

‡NA, not available.

We used European Centre for Disease Control (ECDC) data prior to the date of implementation of suppression or, in the absence of identified suppression measures, the date of last entry within the ACAPS dataset. We then evaluated the ratio of reported cases to deaths in this period to provide a measure of the capacity of countries to contain transmission through testing-based approaches prior to, or in the absence of, suppression. Our estimates show clear differences by region and gradient across income strata: LICs with three or more deaths prior to suppression reported a country-level median of 8.6 cases per reported death (country-level range of 7.8 to 10.7, *n* = 3 countries in total) and LMICs, a country-level median of 19.3 (country-level range 9 to 80, *n* = 7 countries in total). By contrast, HICs reported a country-level median of 72.6 (country-level range of 9 to 325.2, *n* = 27 countries in total) cases per reported death. The extent to which reported case-to-death ratios are a reliable indicator of relative case or infection (including asymptomatic) ascertainment rates will depend upon trends in IFR, the reproduction number *R*_0_, and the extent to which deaths are reported, all of which are liable to vary across income strata. However, this trend is suggestive of the extent to which testing capacity will need to be developed in LIC and LMIC settings if approaches such as case identification coupled with contact tracing are to form part of a successful mitigation or exit strategy.

**Fig. 5:**
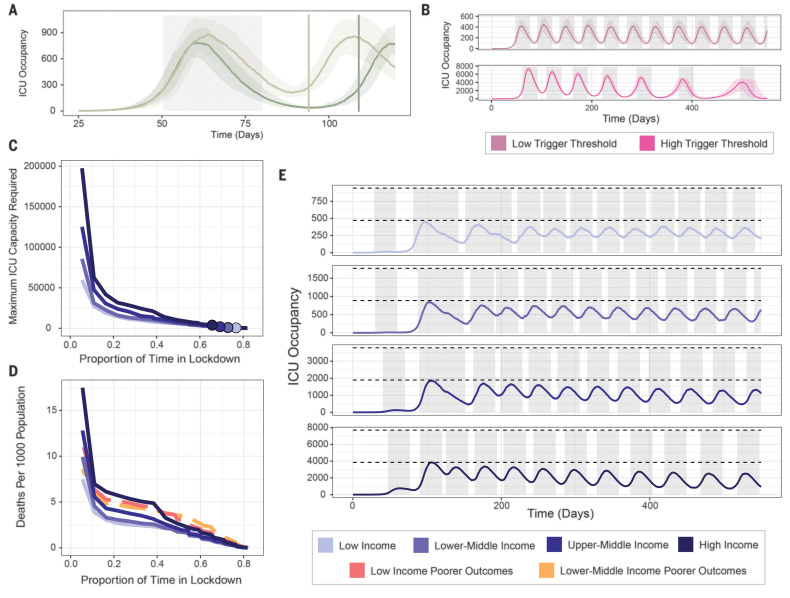
The proportion of time that countries will need to spend in lockdown to remain within health-system critical care capacity. Scenarios are generated using the stochastic SEIR model (see materials and methods). (**A**) The time period between lockdowns for a representative LIC setting, and how it varies with the extent of suppression during lockdown. Gray-shaded area denotes time period of first suppression (triggered at a threshold of 60 ICU cases per day), and brown (75% reduction) and green (85% reduction) vertical lines indicate the next time point at which suppression would be implemented (using the same threshold). (**B**) Time under suppression over the next 18 months for triggering thresholds of 30 (pale pink) and 500 (brighter pink) ICU cases per day, respectively. Gray-shaded areas indicate time in suppression (a 75% reduction in *R*_0_). (**C**) The proportion of time required to be spent in lockdown over the next 18 months as a function of the maximum ICU demand for a representative LIC, LMIC, UMIC, and HIC (colored purple lines). Colored points indicate the median ICU capacity for each of these different income strata. (**D**) The proportion of time required to be spent in lockdown over the next 18 months as a function of the number of deaths caused by the COVID-19 epidemic for a representative LIC, LMIC, UMIC, and HIC assuming comparable quality (but not quantity) of health care across all settings (colored purple lines), and when assuming a reduction in the quality of health care available in LICs and LMICs (red and orange dashed lines, respectively). (**E**) Modeled COVID-19 epidemic trajectories over the next 18 months for a representative LIC, LMIC, UMIC, and HIC where suppression is implemented at ICU incidence trigger thresholds to keep the maximum ICU demand beneath 50% of ICU capacity. The first triggering of suppression has been determined on the basis of the actual patterns of suppression timing observed across LICs, LMICs, UMICs, and HICs.

Given that our estimates suggest that even optimal mitigative strategies will lead to substantial excess mortality and exceedance of health care capacity, we explored the impact of potentially different suppressive strategies, accounting for the interventions that countries have implemented to date. Here we define “suppression” as reducing transmission to a level for which *R*_t _< 1. To do so, we model a 75% reduction in contact rates across all age groups, giving *R*_t _= 0.75 for *R*_0 _= 3.0. We explored different trigger thresholds on the basis of the incidence of cases requiring critical care per 100,000 population) for the implementation of transmission reductions and modeled these as lasting for 30 days before being lifted (typical of the duration of lockdowns occurring to date). Reimplementation then occurs if the trigger threshold is eclipsed again. The level of reduction in contact rates will determine the speed at which the infected population is depleted during the intervention (*24*). Thus, for a fixed period of intervention (assumed here to be 1 month), either starting earlier (i.e., at lower levels of infection) or suppressing to a greater extent (i.e., higher reductions in contact rates) will mean that interventions can be relaxed for longer before the trigger for reimplementation is reached (Fig. 5A). Equally, if a degree of suppression is maintained during the period of relaxation (for example, a 30% reduction in contacts during relaxation compared to 75% during suppression), then the periods in suppression will be shorter, as the reduced *R*_t_ during relaxation will mean that it takes longer to trigger the suppression threshold. Furthermore, a greater health benefit (in terms of reducing cases or deaths) will be achieved for lower trigger levels; however, this is balanced by a slower buildup of herd immunity such that the interventions would need to remain in place for longer in the absence of a vaccine (Fig. 5B).

To further understand how such a strategy might differ between LICs and LIMCs, and between UMICs and HICs, we explored the full range of incidence thresholds that would allow ICU demand to be kept below 50% of the median ICU capacity for each income strata (setting this threshold lower than maximum capacity to allow for ongoing care provision for non–COVID-19–related disease). For each scenario, we modeled the first suppression to have been initiated at the median threshold for the setting that has been observed to date (i.e., LICs and LMICs initiate earlier than UMICs and HICs, Table 1). We then selected the ICU incidence trigger within this range that minimizes COVID-19 mortality over 18 months and remains under our ICU threshold. We use a time window of 18 months as representative of the time scale over which pharmaceutical interventions (e.g., a vaccine) may become available, noting that this duration is highly uncertain (*25*).

If ICU demand is to be kept below the estimated median ICU capacity for each income strata, all countries are predicted to need to spend a substantial proportion of time in suppression (Fig. 5C). In all settings, in the absence of additional effective measures, we estimate that the time spent in suppression to prevent health services from becoming overwhelmed will need to be high across all settings, and marginally higher in LICs (77% versus 66% in HICs over an 18-month time frame), driven by the lower threshold at which suppression has to be reapplied coupled with the resulting less rapid acquisition of immunity. Assuming identical quality but not quantity of care across all settings, the mortality rate under suppression is predicted to be highest in HICs across all suppression threshold triggers considered (and by extension, time in suppression over the next 18 months) because of their older populations (Fig. 5D, purple lines). However, once we incorporate our estimates of the poorer quality of care in LICs and LMICs, we predict a similar number of deaths in LICs and LMICs compared to HICs across all suppression triggers. Thus, in all settings, sustained periods of suppression over the next 18 months are predicted to be required if ICU demand is to be kept below capacity and large levels of excess mortality are to be averted. Conversely, we also estimate that the risks of not maintaining suppression are likely to be similarly high across all settings. If suppression is not maintained (and hence the proportion of time spent in lockdown over the next 18 months is low), then our results suggest a lower per-capita level of burden in LICs and LMICs because of the younger population. However, the uncertainty in our estimates of the quality of care in these settings could mean that this result is reversed (Fig. 5D).

Our estimates of increasing time between suppression triggers in HICs in Fig. 5E assume that there is durable immunity to reinfection; this remains uncertain (*16*). However, given the very low levels of population immunity in LICs and LMICs at 18 months under this assumption, our results indicate that in these settings, measures would have to remain in place well beyond the time window of our simulations in the absence of a vaccine to achieve herd immunity, or equivalent effective exit strategy able to maintain control of the epidemic for values of *R*_t_ that remain close to *R*_0._

However, these results do not account for other interventions that could be implemented during periods in which suppressive measures are not in place. Once the number of new infections drops to a manageable level, it is likely that more widespread testing and isolation of cases coupled with contact tracing can help to prevent a resurgence of transmission, as has been observed in countries such as South Korea (*26*–*28*). However, given the low reported case to reported death ratios in LICs and LMICs during the early stage of the pandemic, for such strategies to be successful, it is likely that support to enhance surveillance will be required to increase infection ascertainment rates substantially. Such testing strategies could be supplemented by the additional use of technology, including digital apps (*29*). The appropriateness of these different interventions will be context specific, and hence it is likely that each country will need to develop strategies based on an understanding of the underlying principles outlined here but adapted to suit its needs.

## Conclusions

The results presented here illustrate the potential impact of the COVID-19 pandemic in LICs and LMICs compared to the epidemics that have occurred to date in UMICs and HICs. Our analyses give insight into how differences in demography, social structure, and health care availability and quality combine and potentially influence the impact of measures that can help reduce the spread of the virus. At the current time, it is not possible to predict with any certainty the exact number of cases for any given country, the precise mortality and disease burden that will result, or the benefits and drawbacks of the different approaches to controlling the virus that are currently being implemented. A full understanding of these will only be available retrospectively.

Although our results illustrate the challenges that many countries will face in attempting to mitigate the impact of local COVID-19 epidemics, it is important to bear in mind that even moderate levels of changes in behavior can avert many infections and hence save millions of lives (*30*). Although suppression will always have the greatest impact on COVID-related morbidity and mortality, the intensity of interventions required needs to be balanced against the wider health risks that diverting all attention to a single disease could entail (*31*, *32*).

It is also important to note that we do not quantify the wider societal and economic impact of the intensive mitigation or suppression approaches; nor do we address the challenge of intensive suppression initiatives in LICs and LIMCs, where a high degree of informal labor makes such interventions challenging and may limit the extent to which they can reduce *R*_t_ below 1 (*33*). These are likely to be substantial, particularly in lower-income countries, where the capacity to provide support for ensuring the livelihoods of the poorest and most vulnerable is most marginal. Moreover, for countries lacking the infrastructure capable of implementing technology-led suppression strategies such as those currently being pursued in Asia (*7*, *27*), and in the absence of a vaccine or other effective therapy, careful thought will need to be given to pursuing such strategies to avoid a high risk of future health system failure once suppression measures are lifted.

Our results highlight the difficult decisions that countries are faced with in the coming weeks and months irrespective of region or income status. Given the likely worse prognosis of severe COVID-19 cases in settings with weaker health systems, coupled with the higher vulnerability of developing economies to the negative effects of stringent NPIs, the trade-offs faced by lower-income countries are complex considering the ongoing uncertainty over the most appropriate and effective exit strategies. In the interim, the priority should be to increase the availability of oxygen support to mitigate the health impact alongside enhancing the capacity for surveillance and wide-scale testing to reduce the spread of infection and tailor appropriate NPIs. In the longer term, ensuring equitable provision of pharmaceutical interventions to lower-income countries once they are developed should be a global priority.

Our analysis demonstrates the extent to which countries have mobilized to combat the COVID-19 pandemic. Many lower-income countries have acted while transmission remains at low levels which is likely to have substantially slowed the spread of the virus. In the absence of a vaccine, all governments are likely to face challenging decisions around intervention strategies for the foreseeable future. However, the still relevant counterfactual of a largely unmitigated pandemic clearly demonstrates the extent to which rapid, decisive, and collective action remains critical to save lives globally.

## Supplementary Materials

science.sciencemag.org/content/369/6502/413/suppl/DC1 Materials and Methods Figs. S1 to S6 Tables S1 to S5 References (*36*–*61*) MDAR Reproducibility Checklist Data S1
